# Nanotechnology based approaches for detection and delivery of microRNA in healthcare and crop protection

**DOI:** 10.1186/s12951-018-0368-8

**Published:** 2018-04-13

**Authors:** Vrantika Chaudhary, Sumit Jangra, Neelam R. Yadav

**Affiliations:** Department of Molecular Biology, Biotechnology and Bioinformatics, CCS Haryana Agricultural University, Hisar, 125004 India

**Keywords:** miRNA, Nanotechnology, Nanomaterials, Disease control, miRNA detection and delivery, Therapeutics, Plants, Animals, Humans

## Abstract

Nanobiotechnology has the potential to revolutionize diverse sectors including medicine, agriculture, food, textile and pharmaceuticals. Disease diagnostics, therapeutics and crop protection strategies are fast emerging using nanomaterials preferably nanobiomaterials. It has potential for development of novel nanobiomolecules which offer several advantages over conventional treatment methods. RNA nanoparticles with many unique features are promising candidates in disease treatment. The miRNAs are involved in many biochemical and developmental pathways and their regulation in plants and animals. These appear to be a powerful tool for controlling various pathological diseases in human, plants and animals, however there are challenges associated with miRNA based nanotechnology. Several advancements made in the field of miRNA therapeutics make it an attractive approach, but a lot more has to be explored in nanotechnology assisted miRNA therapy. The miRNA based technologies can be employed for detection and combating crop diseases as well. Despite these potential advantages, nanobiotechnology applications in the agricultural sector are still in its infancy and have not yet made its mark in comparison with healthcare sector. The review provides a platform to discuss nature, role and use of miRNAs in nanobiotechnology applications.

## Background

Nanotechnology, an emerging field of research encompassing the creation and usage of materials at nano scale, at the interface of physical and biological sciences using either top-down approaches or bottom-up assembly. In the biological domain a huge number of well-organised structures and nanomachines made up of macromolecules have been designed to carry out various biological functions. Their fascinating make-up have encouraged many biometric designs due to unique characteristics of DNA, RNA and proteins at nano scale, these can be a platform for the development of nano structures and devices. The idea was pioneered 30 years ago by developing nanostructures using DNA, leading to explosion of information in the well-recognised field of DNA nanotechnology [1]. Nowadays, RNA molecules have gained interest because of their amazing diversity with respect to structure and function. The packaging RNA (pRNA) molecule can be linked with several therapeutic aids including aptamers, ribozymes, siRNA (small interfering RNA) and miRNA [2]. These outcomes have built a way for RNA nanotechnology to develop novel therapeutic methods for treatment and diagnosis of various type of tumours, viral infections and genetic diseases in humans, animals and plants.

The miRNA is endogenous non-coding RNA, which recently has been found to play a key role in regulatory functions responsible for gene expression in all the organisms. Typically, miRNA is 18–23 nucleotides in length and plays an important role in post transcriptional regulation. These are active and dynamic in cytoplasm, in spite of the fact that their synthesis and transcription takes place in the nucleus. The miRNA profile is changed in diseased state making miRNAs a great target for drug therapy through RNA nanotechnology. Restoration of down-regulated miRNA or restraint of overexpressed miRNA to return miRNA to its ordinary state is the premise of miRNA based disease control which can be additionally investigated to control and manage other various cellular functions also. The therapeutic potential of miRNAs was first realized with the discovery that downregulation of miR-15 and miR-16 is associated with development of B cell leukaemia [3]. Since then these small non-coding RNA have drawn attention not only in biomedical research and drug development but in crop protection also as the expression level of key genes can be controlled by manipulation of cellular miRNAs. This review focuses on current knowledge on miRNA based detection and treatment of various disease using RNA nanotechnology. It also highlights the future prospects of various strategies of RNA nanotechnology to be applicable in plant science together with major obstacles that need to be overcome for successful application.

## Biogenesis and mechanism of action of miRNA

The biogenesis and maturation of miRNA requires different strides which includes nuclear processing, nuclear export and cytoplasmic processing. RNA polymerase II first transcribes the miRNA genes leading to the formation of long primary transcript having a hairpin structure [4–6]. The process of miRNA maturation is initiated by Drosha, cleaving the local stem-loop structure, releasing a ~ 65 nucleotides long hairpin structure (pre-miRNA) [7, 8]. Pre-miRNA is translocated to cytoplasm immediately after Drosha processing, where maturation takes place. A transport complex is formed by protein exportin 5 (EXP5), RAN-GTP, a GTP binding nuclear protein and pre-miRNA [9–13]. Upon export to the cytoplasm, pre-miRNA is cleaved by Dicer near the terminal loop, liberating a small duplex [14–16]. The RNA-induced silencing complex (RISC) is formed by loading of small RNA duplexes generated by Dicer onto AGO (Argonaute) protein [17–22]. After loading of miRNA duplex, the pre-RISC rapidly removes the passenger strand and generates mature RISC. This binds to the 3′-untranslated region (UTR) of the target mRNA based on a partial miRNA-mRNA complementarity leading to translational inhibition and/or degradation of the target mRNA.

Plant miRNAs (highly variable in length) are generally transcribed by RNA pol II in a way similar to animals [23–25]. However, unlike animals, processing of plant pre-miRNA occurs in nucleus and homologues of Drosha and DGCR8 are not reported. Most of the processing is done by DICER-LIKE 1 protein by sequential cleavage. DCL 1 (DICER-LIKE 1) interacts with DAWDLE (DDL), an RNA binding protein and stabilizes pre-miRNA nuclear foci called D-bodies (dicing bodies). A complex is formed by SERRATE (SE), a zinc figure protein, HYPONASTIC LEAVES 1 (HYL1) a dsRNA binding protein and nuclear cap-binding complex and process pre-miRNA [26–28]. After processing, HUA ENHANCER1 (HEN1) the 3′end of the miRNA passenger strand is methylated at 2′-O, which blocks uridylation by HEN 1 SUPPRESSOR [26, 28] and decomposition of miRNAs by SMALL-RNA DEGRADING NUCLEASE 1, a 3′–5′ exoribonuclease [28–30]. After processing, HASTY (HST) export mature miRNA to cytoplasm [26].

## Expression pattern of miRNA in diseased state

MiRNAs are involved in various biological processes such as cell growth and development, biogenesis, signal transduction and apoptosis. MiRNA genes are frequently subjected to genetic alterations as they are located in fragile genomic regions. The epigenetic changes can bring about varied miRNA expression prompting to over expression or down regulation of miRNA leading to various diseases like cardiac, diabetes, neurological and many more. MiR-145 is differentially expressed in various types of cancers like colon, breast, lung and prostate [31–35].

A large number of miRNAs have been reported in plants, which are evolutionary conserved and regulate several biological processes. These are also found to be associated with various bacterial, fungal and viral diseases. These miRNAs are differentially expressed in growth and development as well in response to biotic and abiotic stress. The miR156 and miR159 are dominant families which are associated with pathogen response in plants and can be targeted for controlling serval bacterial, fungal and viral diseases in plants. These miRNA families may provide an excellent opportunity for engineering disease resistance for crop protection. For example, differential expression of miRNA has also been observed in diseased plants. Twelve miRNAs (miR156, miR159, miR160, miR164, miR166, miR168, miR172, miR319, miR398, miR408, miR1448 and miR1450) were found upregulated in stem bark of *P. trichocarpa* when infected with stem canker pathogen [36]. Many highly conserved miRNAs (miR156, miR159, miR160, miR162, miR166, miR167, miR169, miR172, miR319 and miR396) were downregulated upon infection with *Verticillium dahlia* in Eggplant [37]. Gao et al. found that in the plants infected with HCRV (Hibiscus chlorotic ringspot virus), the level of four conserved miRNAs (miR171, miR168, miR167 and miR165) increased with the increase in sulphite oxidase (SO) level [38]. Wu et al. for the first time showed that miR811 and miR829 confers a high degree of resistance to *Exserohilum turcicum* in maize. They also found that miR811, miR829, miR845 and miR408 were differentially expressed in infected plants [39]. Aberu et al. reported that the expression of four miRNAs (miR156, miR162, miR398 and miR408) involved in proteasomal degradation in papaya when infected with *Papaya meleira* virus was increased in response to very low levels of PMeV titre and decreased as the viral titre increases [40]. Twenty miRNAs (osa-miR812j, osa-miR5072, osa-miR444a-5p, osa-miR396c-3p, osa-miR1870-5p, osa-miR1863b.2, osa-miR172d-5p, osa-miR171c-3p, osa-miR167a-5p, osa-miR166d-5p, osa-miR159b, osa-miR156-3p, osa-miR15a, osa-miR1432-5p, osa-miR1432-p, osa-miR1429-5p, osa-miR1432-3p, osa-miR1429-5p, osa-miR1425-3p, osa-miR1423-5p and osa-miR1320-5p) are upregulated upon infection with rice stripe virus in susceptible plants [41]. In *Ziziphus jujube*, 12 miRNAs were upregulated two-fold including miR156a, miR156b, miR156c, miR156d, miR156e, miR156 h, miR159e, miR319a, miR395a, miR395b, zju-miRn23 and zju-miRn24. Ten miRNA were down regulated two-fold including miR159a, miR172, miR2111, miR2950, miR399, miR477, miR858b, zju-miRn2, zju-miRn8 and zju-miRn16 in response to Witches’-Broom phytoplasma infection [42]. A decrease in slmiR4828 and slmiR5300 was observed in Motelle plant when infected with *F. Oxysporum* [43]. This differential expression of miRNAs in humans, animals and plants has been analysed for early detection of diseases as discussed in Table 1.

**Table 1:** Up regulation and downregulation of various miRNA in diseased state

Micro RNA	Up/down regulated	Disease	References	
Humans				
miR-660-3p, miR-665, miR-1285-3p and miR-4491, miR-21, miR-29b, miR-34, miR-122, miR-129, miR-142-3p, miR-199, miR-210, miR-211, miR-212, miR-379, miR-423-5p and miR-503	Up	Chronic heart failure	[47–49]	
miR-221-30, miR-487b-3p, miR-4288, miR-30, miR-107, miR-125b, miR-126, miR-139, miR-142-5p, miR-182, miR-497 and miR-526	Down	Chronic heart failure	[47–49]	
miR-133 and miR-208a	Up	Coronary artery disease	[50]	
miR-126, miR-17, miR-92a, miR-145 and miR-155	Down	Coronary artery disease	[50]	
miR-24, miR-21, miR-20b, miR-15a, miR-126, miR-191, miR-197, miR-223, miR-320, miR-486, miR-150, miR-29b, miR-206 and miR-133a	Down	Type-II diabetes	[51, 52]	
miR-28-3p, miR-9, miR-29a, miR-30d, miR34a, miR-124a, miR-146a and miR375	Up	Type-II diabetes	[51, 53]	
miR-150, miR-27a, miR-192, miR-320a and miR-375	Up	Type-II diabetes	[54]	
miR-152, miR-30a-5p, miR-181a, miR-24, miR-148a, miR-210, miR-27a, miR-29a, miR-26a, miR-27b, miR-25 and miR-200a	Up	Type-I diabetes	[55]	
miR-21a, miR-93, miR-31, miR-9, miR-34a, miR-155, miR-181a and miR-199a	Down	Type-I diabetes	[56, 57]	
miR-29a, miR-122, miR-132,	Down	Gestational diabetes mellitus	[58]	
miR-29aand miR-92a	Up	Colorectal cancer	[59]	
mir-009-1, mir-155, mir-213 and mir-102	Up	Breast cancer	[60]	
mir-010b, mir-021, mir-034, mir-123, mir-125a, mir125b-1, mir-140-as, mir-145, mir-194 and mir-204	Down	Breast cancer	[60]	
miR-200a, miR-200b, miR-200c, miR-141, miR-205, miR-21 and miR-182	Up	Ovarian cancer	[61]	
mir-124a, mir-211, mir-216, mir-302c, mir-145, mir-29c, mir-181a, mir-302, let-7c, mir-98, mir-19b, mir-29b, mir-18a, mir-154, mir-19a, mir-153, mir-144, mir-222, mir-302a, mir-204, mir-101-1, mir-214, mir-34c, mir-374, mir-195, mir-215, mir-28, mir-29a, mir-133b, mir-299-5p, mir-7-2, let-7f, mir-139, mir-193, mir-323, mir-134, mir-370, let-7d, mir-100, mir-101, mir-105, mir-125a, mir-125b-1, mir-126, mir-133a, mir-137, mir-140, mir-143, mir-147, mir-199a, mir-199b, mir-224, mir-302b, mir-9 and mir-99a	Down	Ovarian cancer	[61]	
miR-20, miR-21, miR-99a, miR-141, miR-182, miR-198, let-7a, miR-148a, miR-200c, miR-375, miR-96, miR-141, miR-145, miR-155, miR-34, miR-34c, miR-187, miR-221 and miR-224	Up	Prostate cancer	[62–66]	
miR-145, miR-155, miR-143, miR-223, miR-149, miR-181b, miR-205, let-7a and miR-182	Down	Prostate cancer	[62–66]	
miR-30e, miR-181b, miR-34a, miR-346 and miR-7	Up	Schizophrenia	[67]	
miR-151a-3p, miR-181b-5p, miR-320a, miR-328, miR-433, miR-489, miR-572 and miR-663a	Down	Autism	[68]	
miR-101-3p, miR-106b-5p, miR-19b-3p, miR-195-5p, miR-130a-3p and miR-27a-3p	Up	Autism	[68]	
miR-98-5p, miR-885-5p, miR-483-3p, miR-342-3p, miR-191-5p and miR-let-7d-5p	Down	Alzheimer’s	[69]	
miR-194-5p, miR-301a-3p, miR-30b-5p, miR-342-5p and miR-4446-3p	Down	Drug resistant epilepsy	[70]	

Micro RNA	Animal	Up/down regulated	Disease	References
Animals				
bta-miR-15b, bta-miR-17-3p, bta-miR-16b, bta-miR-148a, bta-miR-26b, bta-miR-101, bta-miR-29b, bta-miR-27b and bta-miR-215	Cow	Up	Bovine metritis	[71]
bta-miR-148b, bta-miR-199a-3p, bta-miR-122, bta-miR-200b and bta-miR-10a	Cow	Down	Bovine metritis	[71]
mir-19b	Pig	Down	PPV infection	[72]
bta-mir-100	Cow	Up	*S. uberis* infection	[73]
bta-mir-19b, bta-mir-19b-2, bta-mir-1271, bta-mir-100, bta-mir-301a, bta-mir-32, Novel-14_7917	Cattle	Down	Paratuberculosis	[74]
bta-mir-6517, bta-mir-7857	Cattle	Up	Paratuberculosis	[74]
bta-miR-24-3p	Cattle	Up	*Mycoplasma bovis* infection	[75]
bta-miR-92a, miR-92a	Cattle	Down	*Mycoplasma bovis* infection	[75]

Micro RNA	Plant	Up/down regulated	Disease	Reference
Plants				
vvi-miR395a-m, vvi-miR398b-c, vvimiR408, ccamiR408, c31052, c141107, c141224 and c205570	Grapevine	Up	Leaf roll associated virus-3 infection	[76]
miR171	Arabidopsis	Down	Viral infection	[77]
miR156, miR157, miR158, miR162, miR164, miR167 and miR171	Arabidopsis	Up	Turnip yellow mosaic virus	[78]
miR393	Arabidopsis	Up	*P. syringae*	[79]
miR393	Soybean	Up	*P. sojae*	[80]
miR482, miR1448, miR1971 and miR2118	Cotton	Down	Vetricillium	[81, 82]
miR156, miR159, miR164, miR168	Wheat	Down	*Erysiphe graminis* f. sp. Tritici	[83]
miR156, miR159, miR164, miR168, miR169, miR172, miR393, miR396, miR482, miR1448, Phe-SR3, Phe-SR23, Phe-SR25	*Populus beijingensis*	Up	*Dothiorella gregaria*	[84]
miR156, miR162, miR398 and miR408	Papaya	Up	*Papaya meleria*	[40]
miR845 and miR811	Maize	Up	*Exserohilum turcicum*	[39]
osa-miR812j, osa-miR5072, osa-miR444a-5p, osa-miR396c-3p, osa-miR1870-5p, osa-miR1863b.2, osa-miR172d-5p, osa-miR171c-3p, osa-miR167a-5p, osa-miR166d-5p, osa-miR159b, osa-miR156-3p, osa-miR15a, osa-miR1432-5p, osa-miR1432-p, osa-miR1429-5p, osa-miR1432-3p, osa-miR1429-5p, osa-miR1425-3p, osa-miR1423-5p and osa-miR1320-5p	Rice	Up	Rice stripe rust virus	[41]
miR156a, miR156156b, miR156c, miR156d, miR156e, miR156 h, miR159e, miR319a, miR395a, miR395b, zju-miRn23 and zju-miRn24	*Ziziphus jujube*	Up	Witches’-Broom phytoplasma	[42]
miR159a, miR172, miR2111, miR2950, miR399, miR477, miR858b, zju-miRn2, zju-miRn8 and zju-miRn16	*Ziziphus jujube*	Down	Witches’-Broom phytoplasma	[42]
slmiR4828 and slmiR5300	Motelle	Down	*F. oxysporum*	[43]

Interplay of TFs with miRNA is well-known. For example in wheat, interaction between miR164 and a novel transcription factor NAC21/22 is said to be involved with resistance against *Puccinia* [44]. In Populus, TC-rich repeats, MBS and W1-box are three most dominating cis-elements present in fungus responsive miRNA accounting for 92.7, 85.4 and 82.9% of 41 fungi-responsive miRNA molecules [36]. WRKY transcription factors interact with Md-miR156ab and Md-miR395 to induce resistance against leaf spot disease in apple [45]. NF-YA transcription factor and miR169 are involved in *R. solanacearum* pathogenicity in Arabidopsis [46].

## Nanotechnology for miRNA detection

MiRNAs are key governors of altered gene expression under many pathological conditions in plants, animals and humans, and are characterized by altered miRNA levels. Therefore, precise and sensitive assessment of miRNA can lead to early and correct disease diagnosis and make these small noncoding RNA transcripts a valuable biomarker [85]. Conventionally, miRNA detection is based on real-time polymerase chain reaction (qRT-PCR) and microarrays. These techniques are error prone, undergo cross hybridization and need legitimate and valid internal control because miRNA are short sequences making designing of probes and primers difficult [86, 87]. Aiming at overcoming some limitations of conventional quantification techniques, nanotechnology is right choice making sensitive and stringent alternatives for miRNA sensing.

## Nanoparticle based miRNA detection

Gold nanoparticles promise a viable and sensitive detection technology as their optical properties can easily be tuned and can be surface functionalized using a variety of well characterized chemical moieties that is thiols, disulphides and amines [88]. Gold nanoparticles-decorated graphene field-effect transistor (FET) biosensor is highly sensitive, selective and can be used for label-free detection of miRNA. It is a potential technique for diagnosis of genetic diseases [89]. Gold nanoparticle coated magnetic microbeads are used as an immobilization matrix for higher loading density of hairpin-structured probes. Ferrocene (Fc)-capped gold nanoparticle/streptavidin conjugates, amplified electrochemical assay of miRNA has been performed for the detection of miRNA-182 from serum samples of glioma patients [90]. A new class of intracellular nanoprobe, termed AuNP loaded split DNAzyme-probe was developed to sense miRNA in living cells. In the absence of target miRNA, the split DNAzymes form an inactive DNAzyme motif with their substrate through partial pairing at the end of each strand and the fluorescence is quenched. Inside the cells, the target miRNA binds with both of the two halves of split DNAzymes forming the active secondary structure in the catalytic cores which can cleave the substrates resulting in the rupture of the substrate and recovery of fluorescence [91]. A dual-mode electronic biosensor was developed for miRNA-21 detection based on gold nano-particle-decorated MoS2 nano-sheet which may prove to be potential candidate in biosensing field from nucleic acid to protein detection [92]. A sensor for the detection of specific miRNA sequences was developed based on graphene quantum dots (GQDs) and ssDNA-UCNP@SiO_2_ [93]. Carbonaceous nanomaterials such as nanosized graphene oxide and carbon nanoparticles have also been used in miRNA detection which is based on their fluorescence quenching properties [85]. The identification and detection of miRNAs has profound implications in cancer therapy [94] and nanoparticles can assist in development of novel disease treatment strategies.

## Nanopore based miRNA detection

Nanopore provides a sore single molecule platform for digging into a large variety of life science troubles [95–97]. Nanopore sequencing applications have gained momentum and the technology has become cheaper. Nevertheless, nanopores have the capacity to detect epigenetic changes in a DNA sequence. Nanopore can detect the position and conformation of a single molecule with high specificity that is present in pore lumen. By the particular conductance change in nanopore, one can electrically measure and elucidate single-molecule kinetic pathways in the target. In light of biotechnological applications, different nanopore sensors have been produced [98, 99]. In the today’s quickly advancing technology, development of nanopore based miRNA detectors is a novel exertion. A 3 nm synthetic probe was used for the first time to quantify the translocation of enriched miRNA that was hybridized to a probe [100]. They developed a nanopore based miRNA detector for lung cancer that produced signature electric signs using a programmable oligonucleotide probe for direct and label free identification of target miRNA in wavering surrounding. The key segment of the nanopore detector is the probe whose sequence is programmable and can be improved to accomplish high sensitivity and selectivity. Specific chemical modification can be employed to engineer the probe, so that simultaneous miRNA detection can be done using distinct probe [97, 98]. This sensor can be utilized for the detection of miRNAs, nucleic acid fragments [101, 102], genetic alterations and pathogenic DNA/RNAs. High throughput detection can be done by utilizing a newer membrane platform droplet interface bilayer [103]. Sub-picomolar levels of miRNAs can be quantified using this technique [104] and nanopore-based miRNA detection techniques will be further improved and validated for disease control in coming future [105].

## MiRNA therapeutics

Despite the fact that we have just barely started to pick up bits of knowledge into the full scope of biologic elements of miRNA, their inclusion in the onset and movement of diseases has created huge enthusiasm for its use in therapeutics. Mounting proofs recommend that miRNA-based treatments, either by restoring or repressing miRNAs expression and activity hold great promise in therapeutics. The unusual expression of miRNA in various diseases makes it a critically important focus for researcher to create more strategies to develop newer techniques for disease diagnostics and treatment. Biologists have understanding that several different miRNAs are upregulated upon infection with various diseases. Under this situation, antisense inhibitors of the overexpressed mature miRNA sequence can be used to hinder the overexpression as restorative technique. The miRNA expression can be reset to restore the normal cell functioning by providing mature miRNA exogenously. Presently, miRNA activity is regulated by two approaches (i) restoring miRNA function using synthetic double stranded miRNAs, (ii) inhibiting miRNA function using chemical modified anti-miR oligonucleotides [106].

## Restoring miRNA function

The normal functionality of miRNA in the diseased conditions which caused downregulation of miRNA can be regained by two ways [107]. First by direct replacement miRNA equivalent to dicer products using synthetic mimics of the targeted mature miRNA. These engineered mimics are closely resembled siRNA and can be straightforwardly delivered into the cell cytoplasm for loading into RISC complex [108]. The other approach to restoring typical miRNA function is overexpression of specific miRNA utilizing vectors. Short hairpin RNA from polymerase II or III promoters has been expressed and processed by Dicer ahead of RNA loading into RISC [109].

## MiRNA inhibition

When miRNA upregulation leads to disease symptoms, the miRNA inhibition therapy can be employed to regulate the expression. Most methods involve miRISC complex disruption. MiRNA inhibition can be done either by using miRNA sponges or antisense oligonucleotides called anti-miRs. In miRNA sponge, the function of a specific miRNA or miRNA family is blocked by transgenic overexpression of RNA molecule harbouring complementary sites to miRNA of interest [110]. Chemically modified anti-miR oligonucleotides are required for efficient silencing of dysregulated miRNA in vivo due to better binding affinity, biostability and pharmacokinetic properties. The chemical modifications includes phosphorothioates containing oligonucleotides [111], 2′-o methyl-(2′-O-Me) or 2′-O-methoxyethykoligonucleotides [112], locked nucleic acids (LNA) oligonucleotides [113], peptide nucleic acids [114], fluorine derivatives (FANA and 2′F) and other modifications [115]. For improving the duplex melting temperature and enhancing nuclease resistance of anti-miRs sugar modification is most commonly employed. Locked nucleic acids are found to have superior in vivo stability and target binding ability and show the highest affinity towards complementary RNA making use of miRNA as therapeutics a great consideration [116–118]. MiRNA represents a novel and attractive target in terms of scientific perspectives to manipulate body functions. A snapshot of present development status of miRNA based therapeutics is presented in Table 2.

**Table 2:** Present scenario of miRNA therapeutics

Company	Targeted miRNA	Disease	Technology	Status
Regulus Therapeutics	miR-122	Hepatitis C	Anti-miR	Preclinical
	miR-10b	Glioblastoma	Anti-miR	Preclinical
	miR-221	HCC	Anti-miR	Preclinical
	miR-21	Renal fibrosis	Anti-miR	Preclinical
	miR-33	Atherosclerosis	Anti-miR	Completed preclinical
	miR-17	Autosomal dominant polycystic kidney disease	Anti-miR	Preclinical
	miR-27	Cholestatic disease	Anti-miR	Discontinued
	miR103/107	Type-II diabetes	Anti-miR	Phase-I
Santaris Plasma	miR-122	Hepatitis C	Anti-miR	Phase-IIa
Mirna therapeutics	miR-34	Primary liver cancer	Mimic	Phase-I
	miR-155	Hematological malignancies	Anti-miR	Completed preclinical
	miR-215	Cancer	Mimic	Preclinical
	miR-101	Cancer	Mimic	Preclinical
	miR-16	Cancer	Mimic	In pipeline
	let-7	Cancer	Mimic	In pipeline
miRagen therapeutics	miR-92	Peripheral artery disease	Anti-miR	Preclinical
	miR-15/195	Myocardial infarction	Anti-miR	Preclinical
	miR-155	Cutaneous T-cell lymphomas	Inhibition	Phase-I
	miR-208	Chronic heart failure	Inhibition	In pipeline
	miR-143/145	Vascular disease	Inhibition	In pipeline
	miR-29	Cardiac fibrosis	Inhibition	In pipeline
	miR-451	Abnormal red blood cell production	Anti-miR	Discovered
	miR-92	Peripheral arterial disease	Inhibition	In pipeline

## Challenges in miRNA based therapies

With the advancement of RNA innovation, miRNA have drawn extraordinary consideration and their applications in research and pharmaceuticals are being quickly expanded. In addition to repressing gene expression, miRNA can also activate genes in some cases or even bring pseudo activity by themselves.

### Biological instability and short circulation half-life

MiRNA or other RNA-based nanostructures, for instance, anti-miRNA oligonucleotides (AMO) have short half-life and immediately degraded in natural fluids by widespread nucleases. Particularly, nanostructures are more sensitive to natural changes, such as the change from in vitro to in vivo conditions.

### Reduced intracellular delivery

In spite of the fact that pRNA has been shown to be a powerful nano-vector for delivery of miRNA, regardless it faces many difficulties, for example, industry scale synthesis and purification is not all that simple yet.

### Off target effects

MiRNA activity depends on imperfect base-matching with target sequences, which may lead to lower specificity. Also, a miRNA may focus on a few obscure qualities in the meantime and one gene can be regulated by a few miRNAs [119], which indicates that its mode of action is more complicated than anticipated.

## Necessity of nanocarrier-mediated miRNA delivery

Oligonucleotides like siRNA, anti-miRNA and synthetic miRNA mimics can be linked with nanocarriers which improves their resistance to nucleases [120]. The chances of immune rejection are reduced to many folds when nanocarriers are used to deliver oligonucleotides [121]. The function of nanoparticles can be modulated because nanocarriers can be easily modified with different molecules. For example, nanoparticles can be easily conjugated with proteins, antibodies and carbohydrates by modifying the surface with carboxyl and amino groups [122]. The therapeutic miRNA is delivered with high specificity when targeting ligand is attached on the nanoparticle surface [123]. Many obstacles are faced by miRNA while reaching its target site the body when delivered naked. So, miRNA can be linked with PEG coated, surface-passivized nanoparticles leading to miRNA protection from degradation, opsonization and enhances circulation. Due to the small size and ability to link cell penetrating peptides of nanoparticles, the cellular uptake of miRNA is enhanced resulting in many fold rise in cellular entry [124].

## Nanotechnology-assisted miRNA delivery

In the recent years, nanotechnology has been through many advancements both in terms of novel material development [125] and applications in growing number of areas. These advancements have led to the development of novel DNA and RNA delivery systems for disease control that can be used instead of viral vectors. Variety of inorganic (gold, silver, calcium phosphate, graphene, quantum dots, iron oxide and silica), organic (chitosan, liposomes, proteins/peptides and aptamers) and polymers based nanomaterials can be used for the development of these non-viral vectors. These nanomaterials based delivery systems have several advantages over viral vector based delivery systems, which include decreased immune response and flexibility in design which allows them to be functionalized and targeted to specific sites with low cytotoxicity [126].

## Chitosan based nanomaterials

Chitosan is a natural polysaccharide of β-1-4 linked *N*-acetyl-d-glucosamine and d-glucosamine. It is generally produced by deacetylation of chitin from crustacean shells. Due to its cationic nature, natural abundance, low toxicity, biodegradability and mucoadhesivity [127, 128] it has been used to as in vitro vector for DNA, [129–131] and miRNA delivery. McKiernan et al. targeted cystic fibrosis using miRNA based nano-medicine. They used cytosine and PEI based nano delivery method to target miR-126 [132]. Deng et al. reported that hyaluronic acid-chitosan nanoparticle mediated delivery of miR-34a with doxorubicin, suppresses breast cancer [133]. Louw et al. found that chitosan mediated delivery of miRNA-124 reduces microglial cells in spinal cord injury in rat [134].

## Lipid/liposomes based nanomaterials

Liposome mediated DNA/RNA/miRNA delivery is usually done with the help of cationic lipids (CLs) [135, 136]. These lipids comprise a cationic head group and a linker to which a hydrophobic moiety is attached. The negatively charged phosphate group of the nucleic acids binds to the positive charge of head group [137]. Liposomes own attributes like less toxic, easy to handle and reduced risk of immunological rejection, which makes it a handy tool for using in non-viral DNA delivery [138, 139]. Lactosylated gramicidin based lipid nanoparticles were used to deliver anti-mir-155 to hepatocellular carcinoma [140]. Hsu et al. used cationic lipid nanoparticles to deliver miR-122 mimic to downregulate miR-122 in hepatocellular carcinoma [139]. Pramanik et al. used lipid based nanoparticles to deliver mimic miR-34a or miR-143/145 to inhibit pancreatic cancer in mice [141]. Wu et al. delivered miR-29b or miR-133b using cationic lipoplex based vectors for the treatment of lung cancer [142]. Piao et al. used lipid based nanoparticles to deliver pre-miR-107 to inhibit tumorigenicity of head and neck squamous cell carcinoma [143]. Liu et al. used liposome based nanoparticles to deliver anti-miR-296 for inhibiting blood vessel formation in cancer cells [144].

## Protein and peptide nanomaterials

Nucleic acid carriers designed with the help of proteins and peptides are widely explored to deliver nucleic acids and small non-coding RNA molecules because the nucleotides are condensed through electrostatic interactions with the positive charge of amino acids. Furthermore, amino acids add to endosomal escape, targeted delivery and bioreversible polyplex stabilization of system [145]. Oh et al. studied the effect of R3V6 peptide mediated delivery of anti-miR-21 in glioblastoma in animals [146]. Song et al. studied the effect of peptide mediated delivery of anti-miR-21 in glioblastoma [147]. Suh et al. studied the anti-cancer effects of peptide mediated delivery of miR-29b [148]. Peptide based nanocomplexes were developed to deliver anti-miR-199a to inhibit pancreatic cancer [149].

## Aptamers

Aptamers are 3-D (three-dimensional) single chain DNA or RNA molecules that bind to a particular target molecule with high affinity and particularity [150]. Repeated rounds of selections known as “systematic evolution of ligands by exponential enrichment” (SLEX) is used to select aptamers from a combinatorial library of randomized sequences [151]. Aptamers can be used to target small molecules, nucleic acids, proteins, carbohydrates, whole cells and even organism. Aptamers possess high diversity and high specificity to target molecule which can be selected or isolated from the library. In addition to this, aptamers are highly stable and have very low chances of immunogenic rejection. MUC-1 aptamer was used for miR-29b mediated inhibition of PTEN methylation in ovarian cancer [152]. RNA nanoparticles containing anti-prostate-specific membrane antigen (PSMA) RNA aptamers were constructed to deliver anti-miR17 in cancer cells for the treatment of prostate cancer [153]. Systemic delivery of anti-miR-21 using epidermal growth factor targeting aptamer was used to block breast cancer in mouse [154]. RNA adapter GL21.T mediated delivery of miR-212 down regulates PED (apoptosis related protein) and restore TRAIL (TNF-related apoptosis inducing ligand) mediated cytotoxicity in lung cancer [155]. DNA aptamer based anti-human KIT sequences and mouse c-KIT aptamer targeted delivery of miR-26a protects mice from chemotherapy toxicity while suppressing tumour growth [156].

## Inorganic nanoparticles as carrier of miRNA

Inferable from nano-measurement size to volume proportion and its strength, inorganic (metal) nanoparticles are as a rule broadly utilized as promising quality bearers in different biomedical applications. Inorganic nanomaterials have remarkable electrical and optical properties, biocompatibility and in addition low cytotoxicity [157].

### Gold nanoparticles

Among the different metals, suitability of gold as nanoparticles is highest due to its inactive nature, ease of synthesis, high functionalization, ability, higher absorption coefficient, simplicity of location and potential ability of targeted capacity. Therefore, it is widely utilized for different applications including medication and gene delivery. Due to its remarkable stability, large surface area, surface modification and high biocompatibility gold nanoparticles can retain the native structure and enzymatic activity of the attached proteins or enzyme in the drug delivery. The instability and poly anionic nature of naked miRNAs impede efficient cellular uptake and reduce half-life. Viral delivery requires cloning miRNA mimics/inhibitors requiring costly modifications and both require toxic lipofection or electroporation. To address these challenges, unmodified miRNAs were delivered using cysteamine-functionalized gold nanoparticles (AuNPs) showing highest payload, lowest toxicity, fastest endosomal escape and increased half-lives [158]. Gold nanoparticles functionalized with synthetic miRNAs were able to deliver miRNA to prostate cancer cells [159]. In prostate and breast cancer, miR-145, a well-known tumour suppressor miRNA is strongly downregulated and gold nanoparticles are used for their delivery [160]. Conjugation of gold nanoparticles with miRNA was tested in cell transfection using multiple myeloma cells demonstrating efficient knockdown in the functional luciferase assay [161]. AuNP-miR-375 nanoparticles exhibit high cellular uptake and preserves miR-375’s activities to suppress cellular proliferation, migration and colony formation and to induce apoptosis in hepato-carcinoma cells [162]. Anti-miRNA oligonucleotides which target miR-29b is known to downregulate myeloid cell leukaemia and are effectively delivered utilizing functionalized gold nanoparticles [163].

### Graphene nanoparticle

Graphene is also an attractive nano-materials because of its optical, thermal and electrical properties [164]. The nanoparticles of poly (amidoamine) dendrimers-grafted gadolinium-functionalized nanographene oxide have proven to be an effective carrier for delivering chemotherapeutic drug and gene targeting agents such as miRNA to cancer cells [165].

### Quantum dot nanoparticle

Quantum dots (QD) are semiconductor-based, monodisperse, nanometer-sized crystals [166], which can be synthesized by different methods including colloidal or plasma synthesis mechanism [167]. It was demonstrated that miR-26a-loaded QD nanocomplexes can up-regulate miR-26a expression, arrest cell cycle resulting in the inhibition of cancer cell proliferation in HepG2 cells [168]. It demonstrates that Quantum dot nanocarrier system has potential for early detection, monitoring and localized treatment of specific diseases.

### Silica nanoparticle

Silica has also been used for miRNA delivery. Mesoporous silica nanoparticles have large surface area for functionalization and tunable pore size which makes it novel and ideal biomaterial for different therapeutic applications [169]. These have been used for delivery of miR-122 antagomir and small molecule inhibitors to achieve combination inhibition of its targeted miRNA in Huh7 cells. The first study which demonstrated silica nanoparticle based targeted delivery involved a tumour suppressive and pro-apoptotic miRNA miR-34a to neuroblastoma tumour-bearing mice for the treatment of neuroblastomas [170]. A multifunctional fluorescent nanoprobe with the AS1411 aptamer and a molecular beacon was co-immobilized on the surface of the fluorescent dye-doped silica nanoparticles for target cell specific delivery and intracellular miRNA imaging [72].

## Polymer mediated delivery of miRNA

Both natural and synthetic polymers show variation in their structure, molecular weight together with the chemical properties of the repeat units(s), affect their physico-chemical properties. Due to the large diversity in polymers, polymer based nanomaterials have drawn interest as potential delivery vectors. Polymer mediated delivery provides unique advantage over liposome because of their stability in biological fluids after systemic administration. These are much suitable for industrial scale up as they require less time of preparation. Synthetic polymers include dendrimers, polyethyleneimines and polyphosphoesters.

### Polyethyleneimine (PEI)

It is most extensively studied polymer for use in gene delivery. PEI is utilized for delivery of nucleic acid to the cells because of its buffering limit [171]. It is available as linear PEI or branched PEI. Linear PEI contains all secondary amines while branched PEI contains primary, secondary and tertiary amines. Furthermore, secondary and tertiary amines of the PEI impart buffering capacity. There are few studies which demonstrated the utilization of PEI for miRNA delivery [172–175]. Mir-145 and mir-33a mimics were delivered systemically using PEI-miRNA complex that resulted in significant antitumor effects in colon carcinoma mouse xenograft model [174]. These studies reveal that PEI coated nanospheres have the potential to be used as the novel class of anticancer agents.

### Dendrimers

Dendrimers are mono disperse, nanosized, highly branched macromolecules which are chemically synthesized in a controlled manner to obtain varied surface functionalities. These are important class of polymers used in delivery of oligonucleotides. They have high drug loading capacity either by encapsulation or conjugation. Delivery of mir-7 to human brain glioma cells (U251) using PAMAM dendrimers had higher transfection efficiency and improved therapeutic effects in vitro and in vivo were achieved [176]. The co-delivery of anti-miR-21 using PAMAM dendrimers significantly improved the cytotoxicity of 5-FU, reduced migration and enhanced the apoptosis of U251 glioblastoma cells9 [177]. When mir-126 was delivered to HUVEC cells through poly arginine and RGD (9 arginine-glycine-aspartic acid)-modified dendrimers, it significantly reduced their proliferation and tube formation [178]. Dendrimers achieve the goal of balanced low toxicity and high potency to effectively deliver miRNA.

### PLGA based nanoparticles

Poly(lactic-*co*-glycolic acid) PLGAs are a family of water-insoluble polymers that have been widely used in biomedical applications [179]. These can be readily formed into nanoparticles which entrap biologically active molecules [180, 181]. PLGA particles overcome several limitations which are faced by current miRNA therapy because they can protect nucleic acids from degradation, achieve high loading capacity and different modifications can be applied [182]. In addition, poly(lactic-*co*-glycolic acid) are safe, biodegradable, and they have a long history of success in enhancing the delivery of therapeutic agents. Reduction in tumour growth was observed by polymer (PLGA-b-PEG) mediated delivery of anti-miR-10b and anti-miR-21 in mice [183]. Therapeutic efficacy of anti-miR-21 and gemcitabine co-encapsulated PEGylated PLGA nanoparticles against hepatocellular carcinoma was studied by Devulapally et al. [183]. The effect of co-delivery of doxorubicin and miR-542-3p using hyaluronic acid decorated PEI-PLGA nanoparticle against breast cancer was studied [184]. Apoptotic effects were significantly enhanced by combined delivery of orlistat loaded nanoparticles and doxorubicin or anti-miR-21 loaded nanoparticles using PLGA-PEG-nanoparticles in breast cancer [185]. New insights in neurodegenerative disease (Parkinson’s disease) were gained by the use of miR-124-PLGA nanoparticles [186].

## Silencing miRNA via RNA nanotechnology for targeted therapeutic delivery

The major breakthrough in the field of biological sciences came with the discovery of RNA interference (RNAi) technology in 1998 and has created boulevard for the treatment of various deadly diseases like cancer [187]. In RNAi, a short 21–23 nucleotide double stranded RNA sequence is used to regulate the gene expression. MiRNA and siRNA are two central molecules to RNA interference [188]. While most of the siRNA are artificial sequences, a few endogenous siRNA sequences do occur, but miRNA are endogenous and target multiple genes [189]. RNAi therapy can be used to target any known mRNA sequences that can use to treat any disease that was previously undruggable. RNA nanoparticles are constructed such that the Dicer endonuclease can approach the dsRNA encoding sequences and can generate functional miRNA that can be loaded into RISC (RNA-induced silencing complex) and thereby modulate gene expression by selectively directing mRNAs. Anti-miRNA nanoparticles were effectively used to downregulate a tumour causing miRNAs and increase an endogenous tumour suppressing miRNAs. Triple negative breast cancer growth was inhibited by pRNA-3WJ nanoparticles loaded with EGFR-targeting RNA aptamer and anti-miR-21. Upon systemic injection into mice with breast cancer, RNA nanoparticles harbouring EGFR aptamer effectively targeted and internalized tumour cells via receptor mediated endocytosis [154]. After entering the tumour cells the anti-miR-21 binds to the target region thereby blocking miR-21 tumorigenic properties [190]. In a similar way, pRNA-3WJ nanoparticles loaded with PSMA aptamers and anti-miR-21 were used to target subcutaneous prostate xenografts and were found to inhibit tumour growth in animal models [153].

## MiRNA delivery in plants

Like animals, the RNA nanoparticle based miRNA delivery technique can be utilised in plants to target those biotic and abiotic stresses against which there is no effective strategy for combating them. RNA nanotechnology may revolutionize the crop improvement programmes which will be a step towards food security worldwide. The artificial microRNA (amiRNA) technology can target endogenous miRNA precursors to give rise small RNA guiding gene silencing in plants and animals [191–194]. Oligonucleotide substitutions that mimic the intact secondary structure of endogenous miRNA precursors may lead to targeted silencing of desired gene [195, 196]. The amiRNA technique was used for the first time in human cell lines followed by Arabidopsis [192, 194]. The use of amiRNAs short sequences is beneficial over long hairpin mediated silencing because of reduced off-target effects on host genes and reduced risk of binding to non-target pathogen for use in agriculture. Recently plant resistance to various pathogens has been achieved by expression of artificial miRNA in transgenic Arabidopsis plants providing resistance to turnip yellow mosaic virus and turnip mosaic virus. This was developed using amiR-P69^159^ and amiR-Hc-Pro^159^ by Niu et al. [197]. AmiRNA based on Arabidopsis pre miRNA159a was also used to develop transgenic Arabidopsis plants that confers resistance against cucumber mosaic virus [195]. AmiRNA against Hc-Pro of Potato virus Y (PVY) and TGBp1/p25 of Potato virus X (PVX) were designed using *Arabidopsis thaliana* miRNA159a, miRNA167b and miRNA171a as backbone. Transgenic tobacco plants showed resistance against PVY and PVX [198]. In wheat, resistance against wheat streak mosaic virus (WSMV) was developed using rice miR359 to construct amiRNA precursor, FanGuard (FGmiR395) under a strong constitutive promoter. The transgenic lines showed three types of resistance, i.e. complete resistance, initially immune but resistance break over time and initial resistance followed by plant recovery [199]. Arabidopsis pre-miRNA159a was used to design amiRNA against Watermelon silver motile virus (WSMoV). Six single (A, B1, B2, C, D and E) and two triple (AB1E and B2DC) motif targeting constructs were designed and transformed in *Nicotiana benthamiana.* Triple amiRNA expressing transgenic lines showed complete resistance against WSMoV [200]. Endogenous gene silenced in various plants species using amiRNA technique are listed in Table 3.

**Table 3:** Artificial micro RNA used for disease resistance in plants

Plant species	Disease	amiRNA/Pre-miR backbone	References
Arabidopsis	Turnip yellow mosaic virus and Turnip mosaic virus	amiR-P69^159^ and amiR-Hc-Pro^159^	[197]
Tobacco	Cucumber mosaic virus	amiR-2b	[201]
Tobacco	Cassava brown streak virus	amiR-159a	[202]
Tobacco	Potato virus Y	amiR-HC-Pro	[203]
Grapes	Grapevine fan leaf virus	amiR^CP^-2	[204]
Tomato	Viral infection	amiR-2a/b	[205]
Tobacco	Potato virus Y and potato virus X	amiR-Hc-Pro^159a^ amiR-Hc-Pro^167b^ amiR-Hc-Pro^171a^	[195]
Arabidopsis	Cucumber mosaic virus	amiR-159a	[198]
Cotton	Cotton leaf curl virus	Pre-miR-169a	[206]
Wheat	Wheat streak mosaic virus	Pre-miR395	[199]
Arabidopsis	Water melon silver mottle virus	Pre-miR-159a	[200]
Tomato	Tomato leaf curl virus	amiR-AV1-3	[207]
Arabidopsis	Turnip mosaic virus	amiR^159^-P69	[208]
Rice	Rice stripe virus and Rice black streaked swarf virus	Osa-pre-miR528	[209]
Barley	Wheat dwarf virus	huv-pre-miR171	[210]
Tobacco	Tomato spotted wilt virus	Pre-miR-159a	[211]

## Challenges associated with RNA nanotechnology

Construction of RNA nanoparticle involves coupling of functionalities, module joining, subunit labelling and chemical modification of nucleic acid molecules. Both chemical and enzymatic approaches are utilized in synthesizing RNA building blocks. Although the technology has gained much advancement, still there is a scope for improvement.

### RNA structure prediction

One of the major challenges in RNA nanotechnology is to know the correct structure of the folded RNA nanoparticle. Because of unusual folding properties, the rules that clarify folding of RNA molecules are yet to be classified. With the use of RNA 2D structure prediction programme, only 70% of structure can be predicted accurately [212, 213]. Though various online resources like Mfold, 233 RNA designer, 234 Sfold, 235 NUPACK, 115 nanofolder, 236 and hyperfold, 182 have been developed, prediction of accurate RNA structure and folding remains a major problem. RNA 3D and 4D structure prediction is even more subtle. Advancements in bioinformatics tools have added insights in RNA structure prediction and computing intermolecular interactions but still more refined tools are expected [214–216].

### Stability

RNA in its native form is sensitive to nucleases (RNases). This instability has become a limited factor for its application in nano material construction. With the advancement in technology, several steps have been taken to increase the stability of RNA molecule which includes; chemical modification of bases (5-Br-Ura and 5-I-Ura); alterations in phosphate linking (phosphothioate, boron phosphate) and changes like 2′-F, 2′-O-Me, 2′-NH_2_ on C2′ carbon [217, 218]. RNA stability can also be increased by synthesis of peptide nucleic acids, locked nucleic acids with bridging and 3′ capping [219, 220]. Among all the 2′-fluorine modification is most suitable because its effect on RNA folding and function is minimal [221].

### In vivo half-life and retention time

In vivo retention time of the nanoparticle is determined by its size making it a critical factor in RNA nanotechnology. Scientific reports suggest that 10–100 nm range particles are optimal for non-viral vector based delivery. Particles within this range are large enough to be retained in the body and small enough to bind the cellular receptors and can cross cell membrane [222]. Hence optimal size of RNA nanoparticle is important for its biodistribution and higher retention time for in vivo delivery. The half-life of chemically modified RNA nanoparticles was found to be 5–10 h [223]. So, chemicals modifications have somehow reduced the problem associated RNA nanoparticle half-life and retention time but still, there is a lot of scope for improvement.

### Limited carrying capacity

Presently, RNA nanoparticles have limited carrying capacity and is a major problem associated with today’s RNA nanotechnology. Only the terminal end of the nanoparticle can be labelled with the molecule, particularly of smaller size. Whole chain labelling can increase the carrying capacity of the nanoparticle but may lead to misfolding of the nanoparticle due to steric hindrance ultimately leading to release of the target molecule. Computational methods can be employed to locate the site where the target molecule can be introduced without disrupting the nanoparticle. The carrying capacity of RNA nanoparticles can be increased by constructing dendrimers and RNA dendrimers have been developed up to generation 4 [224]. Dendrimers can increase the carrying capacity because of branched architecture and hence can lead to increased delivery of therapeutic RNA. But over saturation of therapeutic RNA can lead to gene knockdown and unrelated binding and can cause cytotoxicity [225, 226]. Hence, RNA nanoparticles with lower carrying capacity are advantageous in animal studies because of lower toxicity. Thus development of RNA nanoparticles with high carrying capacity and lower toxicity is a big challenge.

### Scaling up

High cost of production and purification of RNA nanoparticles has restricted its use in clinical applications. Typically, RNA nanoparticles are mainly designed within the range of chemical synthesis (maximum of 80 nt). With the advancement in the technology, the cost of oligosynthesis is significantly reduced. Now 10 g of RNA can be synthesized per cycle. However, large scale purification is still a problem. Gel electrophoresis and high-performance liquid chromatography have limited capabilities giving low yield. Recently, ultracentrifugation has been employed for purification of RNA nanoparticles with high yield [227].

### Endosome escape

Receptor mediated endocytosis is one of the way through which RNA nanoparticles can be delivered in human body. Thus, tracking of intracellular RNA nanoparticle is another major challenge with respect to RNA nanotechnology. Similar to endocytosis pathway, RNA nanoparticles first forms the early endosomal vesicle. After sorting, RNA nanoparticles are shifted to late endosomes and then to lysosomes, where these particles get entrapped without reaching the target. An 8 nt, anti-miR LNA fragment was successful in endosomal escape in RNA nanoparticle mediated cancer therapy [154], but the efficacy of non-coding RNA escape from RNA nanoparticle is still a problem as it is found that cancer regression via this method is relatively low [228, 229]. Various tools have been employed to overcome this problem which includes use of acid cleavable linkers like acetyl, hydrazone and maleic amide and use of acid protonating groups like amino esters and sulphonamide [230].

## Conclusion

With the natural occurrence of miRNAs and their role in disease detection and prevention, these small RNAs prove to be a brilliant possibility for use as novel therapeutics. With effective delivery of miRNAs to diseased cells, regulation of specific gene expression and modification of cellular function can be accomplished however successful detection, targeting and delivery of the miRNAs are required. Several distinctive methodologies have been utilised for the delivery of miRNA and different detection procedures are employed using different nanoparticles. However, limitations associated with miRNA delivery can be encountered using RNA nanotechnology, which aims at developing RNA nanoparticles containing a targeting molecule and additionally miRNA. The self-modelling quality of RNA can be used as a powerful technique to design and structure RNA nanoparticles through the interchange of biological, chemical, physical and computational sciences. Inspite of difficulties associated with RNA nanotechnology, this emerging field has an extraordinary application in the field of medicine, biotechnology, synthetic biology and nanotechnology, especially in the field of therapeutics where it can be utilized in the treatment of savage diseases like malignancy and viral ailments. Nanotechnology works with smallest possible particles which raises hopes for improving agricultural productivity. It has also been suggested that artificial miRNA poses fewer biosafety or environmental problems when applied to agriculture compared with transgenic breeding. Nanostructured formulation through mechanisms such as targeted delivery or slow controlled release mechanisms and conditional release, could lead to precision release of active ingredients in response to environmental triggers. RNA nanotechnology in plant science holds promise to detect and control various biotic and abiotic stresses through use of miRNA based biomarkers which will revolutionize crop protection. RNA nanotechnology will help to develop new area of research developing biosensors for forecasting and early detection of plant disease.

## Future outlook

High thermodynamic stability and amazing diversity of RNA and its structure and function holds great promise over DNA Nanotechnology. Since it can be chemically modified, it has been efficiently utilized in human and animal therapeutics, however, plant nanotechnology is still an emerging area of research and its applications in plant disease detection and protection is yet to be seen. Nanotechnology assisted strategies will play key role in integrated pest management supplementing conventional methods in view of environmental concerns. Plant transformation has been used in improving disease resistance in crop plants using overexpression of disease resistance genes, RNAi etc. Synthetic miRNAs can be of particular use and provide excellent choice in these approaches. Delivery of synthetic miRNA in plants is a major concern but recently clay nanoparticles have been used in delivering miRNA for viral protection. The making of synthetic RNA is not cost effective at present; the new developments in this field may lead to novel cost effective strategies. RNA nanoparticles and their use as spray over plants may be advantageous as it is biocompatible, biodegradable and non-toxic making this approach highly flexible and safe.

Nanoparticles synthesized from plants extracts can also be utilized for direct delivery of synthetic miRNA as an efficient method in comparison to Agrobacterium and other direct gene transfer methods in plants. Nanotechnology assisted targeting of miRNA can help in mitigating pests and abiotic stresses under climate change scenario. The miRNAs and transcription factors, being the regulatory molecules, promise to target multigenic traits since these regulatory networks overlap.

Rapid and early detection of miRNA responsible for disease in plants is still a challenging task. The miRNA can be detected using nanoparticle tags in diseased state using miRNA assay. The association of electrocatalytic nanoparticles tags to hybridized miRNA molecules generates measurable current upon adding substrate to the solution providing better detection limit over optical miRNA assay. There could be new innovative approaches as well for miRNA detection and their use in therapeutics in humans and animals and crop protection as well in near future.

## Abbreviations

DNA: deoxyribonucleic acid
RNA: ribonucleic acid
miRNA: microRNA
RNAi: RNA interference
nt: nucleotides
pRNA: packaging RNA
kb: kilo base pairs
GTP: guanosine triphosphate
kDa: kilo daltons
RISC: RNA induced silencing complex
AGO: argonaute
Pol II: polymerase II
amiRNA: artificial microRNA
PLGA: poly(lactic-*co*-glycolic acid)
PEI: polyethyleneimine
siRNA: small interfering RNA
PEG: polyethylene glycol
osa: *Oryza sativa*
zju: *Ziziphus jujube*
